# Phosphoenolpyruvate carboxykinase 2-mediated metabolism promotes lung tumorigenesis by inhibiting mitochondrial-associated apoptotic cell death

**DOI:** 10.3389/fphar.2024.1434988

**Published:** 2024-08-09

**Authors:** Jing Zhang, Wenjuan He, Dongmei Liu, Wenyu Zhang, Huan Qin, Song Zhang, Ailan Cheng, Qiang Li, Feilong Wang

**Affiliations:** ^1^ Department of Pulmonary and Critical Care Medicine, Shanghai East Hospital, School of Medicine, Tongji University, Shanghai, China; ^2^ School of Medicine, Tongji University, Shanghai, China; ^3^ Nanjing Medical University, Nanjing, China; ^4^ Department of Cardiovascular Medicine, Mayo Clinic, Rochester, MN, United States; ^5^ Department of Radiology, Shanghai East Hospital, School of Medicine, Tongji University, Shanghai, China

**Keywords:** phosphoenolpyruvate carboxykinase 2, metabolic reprogramming, lung tumorigenesis, mitochondrial apoptosis, reactive oxygen species

## Abstract

**Background:**

It is unknown how cancer cells override apoptosis and maintain progression under nutrition-deprived conditions within the tumor microenvironment. Phosphoenolpyruvate carboxykinase (PEPCK or PCK) catalyzes the first rate-limiting reaction in gluconeogenesis, which is an essential metabolic alteration that is required for the proliferation of cancer cells under glucose-limited conditions. However, if PCK-mediated gluconeogenesis affects apoptotic cell death of non small cell lung cancer (NSCLC) and its potential mechanisms remain unknown.

**Methods:**

RNA-seq, Western blot and RT-PCR were performed in A549 cell lines cultured in medium containing low or high concentrations of glucose (1 mM vs. 20 mM) to gain insight into how cancer cells rewire their metabolism under glucose-restriction conditions. Stable isotope tracing metabolomics technology (LC-MS) was employed to allow precise quantification of metabolic fluxes of the TCA cycle regulated by PCK2. Flow Cytometry was used to assess the rates of early and later apoptosis and mitochondrial ROS in NSCLC cells. Transwell assays and luciferase-based *in vivo* imaging were used to determine the role of PCK2 in migration and invasion of NSCLC cells. Xenotransplants on BALB/c nude mice to evaluate the effects of PCK2 on tumor growth *in vivo*. Western blot, Immunohistochemistry and TUNEL assays to evaluate the protein levels of mitochondrial apoptosis.

**Results:**

This study report that the mitochondrial resident PCK (PCK2) is upregulated in dependent of endoplasmic reticulum stress-induced expression of activating transcription factor 4 (ATF4) upon glucose deprivation in NSCLC cells. Further, the study finds that PCK2-mediated metabolism is required to decrease the burden of the TCA cycles and oxidative phosphorylation as well as the production of mitochondrial reactive oxygen species. These metabolic alterations in turn reduce the activation of Caspase9-Caspase3-PARP signal pathway which drives apoptotic cell death. Importantly, silencing PCK2 increases apoptosis of NSCLC cells under low glucose condition and inhibits tumor growth both *in vitro* and *in vivo*.

**Conclusion:**

In summary, PCK2-mediated metabolism is an important metabolic adaptation for NSCLC cells to acquire resistance to apoptosis under glucose deprivation.

## 1 Introduction

Apoptosis is a form of programmed cell death which plays a critical role in tissue homeostasis. Of note, cancer cells could acquire the ability to evade apoptosis, which enables their excessive proliferation and survival under stressful conditions (Bertheloot et al., 2021; Attwaters, 2022; Tong et al., 2022). Among various mechanisms that contribute to the evasion of apoptosis in cancer, metabolism reprogramming is emerging as one of key factors (Schiliro and Firestein, 2021; Li et al., 2022). Cancer cells exhibit distinct metabolic signatures from non-malignant cells. Under nutritionally adequate conditions, cancer cells consume much more glucose than normal cells even in the presence of plenty oxygen, a phenomenon called Warburg effect or aerobic glycolysis (Abdel-Wahab et al., 2019). The enhanced glycolysis generates many macromolecular building blocks to sustain a high proliferation rate and metastatic capacity in cancer cells (Dey et al., 2021). During solid tumor progression, however, cancer cells inevitably encounter a nutrient-deprived tumor microenvironment. For instance, the intratumoral glucose level is 3- to 10-fold lower than in normal tissues (Rocha et al., 2015). Nutrient deprivation causes apoptotic cell death in many types of cancer cells (Onodera et al., 2020; Sa-Nongdej et al., 2021). As the major carbon source for cancer cells, glucose deprivation has been recognized as a key inducer of apoptotic cell death (Garlapati et al., 2021; Wang et al., 2022). The impaired glycolysis in cancer cells leads to inadequate production of metabolic intermediates, which are required to meet the cellular demand for the synthesis of many biological macromolecules (DeBerardinis et al., 2008). Moreover, the downregulation of pentose phosphate pathway due to glucose deprivation is unable to produce enough NADPH to sustain reduction–oxidation homeostasis, which ultimately trigger the activation of apoptosis signaling (Moon et al., 2020). However, how cancer cells overcome apoptosis and maintain their capacity to proliferate under glucose-limited conditions remains poorly understood.

Gluconeogenesis is the metabolic process by which organisms generate glucose from noncarbohydrate substrates such as lactate and amino acids. While principally occurs in the liver and kidneys in humans and mice, gluconeogenesis can also be induced to compensate for the decreased glycolysis in several types of cancer cells (Grasmann et al., 2019; Yu et al., 2021; Legouis et al., 2022; Paulusma et al., 2022). Phosphoenolpyruvate carboxykinase (PEPCK or PCK), which is composed of cytoplasmic form (PCK1) and mitochondrial form (PCK2), catalyzes the first rate-limiting reaction in gluconeogenesis (Wang and Dong, 2019). Of note, previous studies demonstrated that PCK2-mediated production of phosphoenolpyruvate (PEP) from glutamine-derived oxaloacetate enables glucose-independent proliferation in non small cell lung cancer (NSCLC) cells (Vincent et al., 2015; Smolle et al., 2020). Moreover, PCK2 is essential for the synthesis of glycerol phosphate which is required for NSCLC cells growth (Leithner et al., 2018). However, it remains unknown the role of PCK2-mediated gluconeogenesis in the evasion of apoptotic cell death in NSCLC cells in the low glucose condition. PCK2 paves a critical way to move TCA cycle carbons to cytoplasm, which has potential impacts on metabolic reprogramming in mitochondria upon glucose restriction (Vincent et al., 2015; Luo et al., 2017; Yu et al., 2021). Therefore, it would be of interest to investigate if and how PCK2-meditated metabolism affects mitochondrial apoptosis induced by glucose deprivation in NSCLC cells.

In the present study, we found that glucose deprivation increases PCK2 expression in NSCLC cells through the induction of endoplasmic reticulum stress (ER stress). Isotope tracing metabolomics showed that PCK2 is a key mediator of gluconeogenesis in NSCLC cells upon glucose restriction. Of note, silencing of PCK2 significantly enhances apoptotic cell death *in vitro* and inhibits NSCLC tumor growth in a xenograft mouse model, indicating that PCK2-mediated gluconeogenesis is critical for NSCLC cells to override apoptosis. Mechanistically, silencing of PCK2 leads to increased burden of the TCA cycle and oxidative phosphorylation, which in turn disrupts the redox balance to induce the activation of Caspase9-Caspase3-PARP signal pathway.

## 2 Results

### 2.1 Glucose restriction increases PCK2 expression through the activation of ER stress signaling in NSCLC cell lines

**FIGURE 1:**
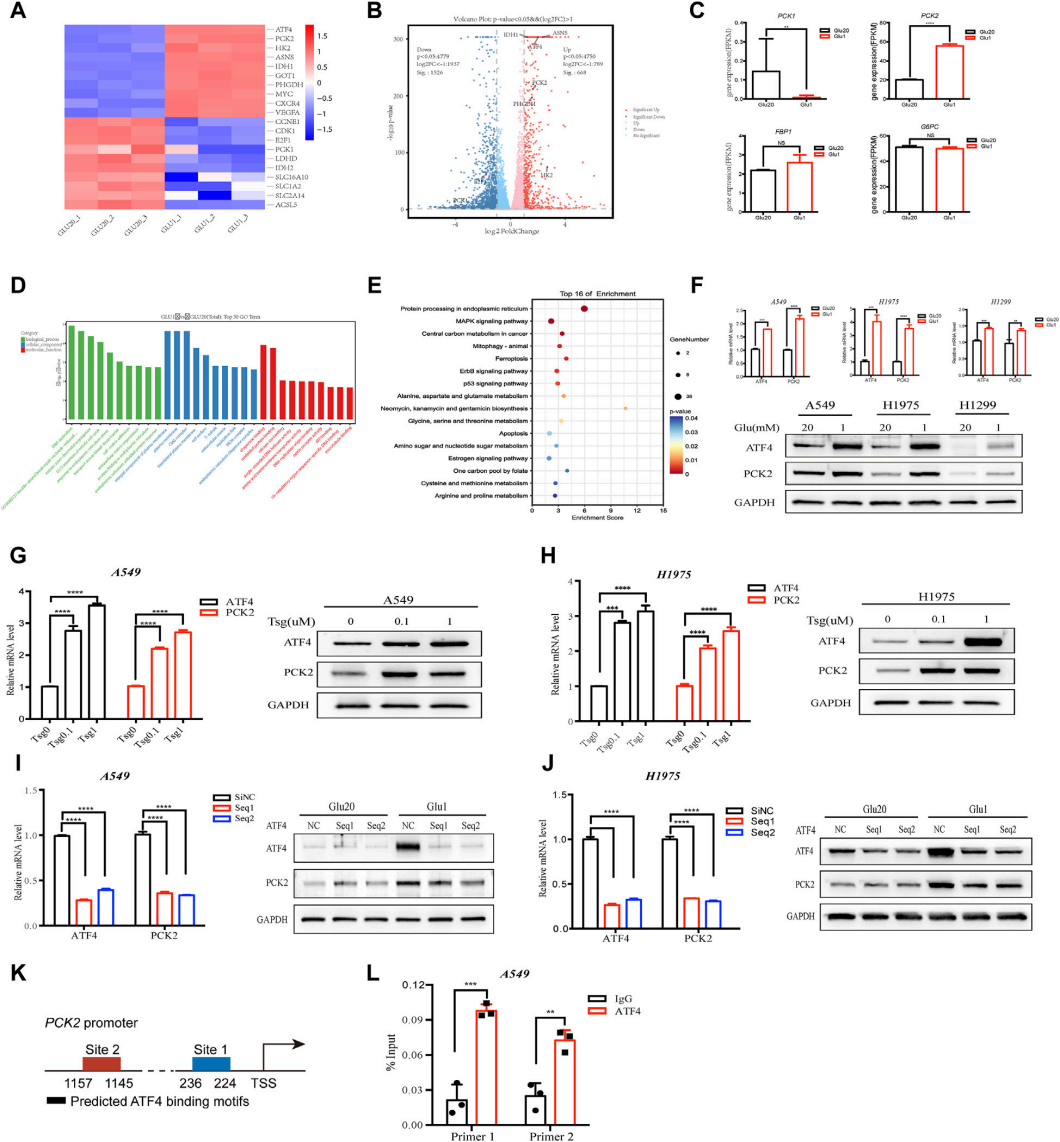
Glucose restriction increases PCK2 expression through the activation of ER stress signaling in NSCLC cell lines. **(A)** Heat map of gene expression in A549 cells cultured in medium containing high (20 mM) and low (1 mM) concentrations of glucose for 24 h. **(B)** Volcano plots of gene expression in A549 cells cultured in medium containing high (20 mM) and low (1 mM) concentrations of glucose for 24 h. FC, fold change. **(C)** Gene expression of gluconeogenic enzymes in A549 cells cultured in medium containing high (20 mM) and low (1 mM) concentrations of glucose for 24 h **p < 0.01, ****p < 0.0001; ns, no significant difference. Data are representative of three independent experiments (n = 3, mean ± SEM). **(D)** GO analysis of differentially expressed genes (DEGs) in A549 cells cultured in medium containing high (20 mM) and low (1 mM) concentrations of glucose for 24 h **(E)** KEGG enrichment analysis of differentially expressed genes (DEGs) in A549 cells cultured in medium containing high (20 mM) and low (1 mM) concentrations of glucose for 24 h. **(F)** Expression of ATF4 and PCK2 mRNA and protein in A549, H1975 and H1299 cells cultured in medium containing high (20 mM) and low (1 mM) concentrations of glucose for 24 h (mRNA) or 48 h (protein). **p < 0.01, ***p < 0.001, ****p < 0.0001. Data are representative of three independent experiments (n = 3, mean ± SEM). **(G)** Expression of ATF4 and PCK2 mRNA and protein in A549 cells treated with different concentrations of thapsigargin for 24 h (mRNA) or 48 h (protein). ****p < 0.0001. Data are representative of three independent experiments (n = 3, mean ± SEM). **(H)** Expression of ATF4 and PCK2 mRNA and protein in H1975 cells treated with different concentrations of thapsigargin for 24 h (mRNA) or 48 h (protein). Data are representative of three independent experiments. **(I)** Expression of ATF4 and PCK2 mRNA and protein in A549 cells transfected with control or ATF4 siRNA for 24 h (mRNA) or 48 h (protein). ****p < 0.0001. Data are representative of three independent experiments (n = 3, mean ± SEM). **(J)** Expression of ATF4 and PCK2 mRNA and protein in H1975 cells transfected with control or ATF4 siRNA for 24 h (mRNA) or 48 h (protein). Data are representative of three independent experiments. **(K)** Two putative ATF binding sites were predicted on the promoter of PCK2 using JASPAR database. **(L)** ATF4 directly bound to the PCK2 promoter. A549 cells were applied to ChIP assay. Data are representative of three independent experiments. **p < 0.01, ***p < 0.001; Data are representative of three independent experiments.

To gain insight into how cancer cells rewire their metabolism under glucose-restriction conditions, RNA-seq was performed in NSCLC cell line A549 cultured in medium containing low or high concentrations of glucose (1 mM vs. 20 mM) for 24 h. As shown in Figures 1A, B, glucose restriction induced a global metabolic adaptation as indicated by the increased the expression of enzymes in several metabolic pathways, including PCK2, hexokinase 2 (HK2), asparagine synthetase (ASNS), isocitrate dehydrogenase 1 (IDH1) and phosphoglycerate dehydrogenase (PHGDH). Of note, the expression of PCK2 rather than other gluconeogenic enzymes (PCK1, Fructose-1,6-bisphosphatase 1, glucose-6-phosphatases) were significantly increased by glucose restriction (Figures 1B, C), indicating that PCK2-mediated metabolism might be required for NSCLC cells to adapt to a low glucose environment. We next explored the underlying mechanisms of how PCK2 expression was induced by glucose deprivation. Gene Ontology (GO) analysis showed that differentially expressed genes (DEGs) were significantly enriched in ER stress-related process, including ER stress response, protein folding in ER and ER stress unfolded protein response, under the biological process category (Figure 1D). In addition, KEGG enrichment analysis of DEGs showed that the protein processing in ER had the highest enrichment score among the top 16 most significant pathways (Figure 1E). These data indicated that glucose-restriction induces ER stress and its related cellular responses in NSCLC cells, which is well-known as the key inducer of transcriptional signaling to promote adaptive cellular responses to stress (Chen and Cubillos-Ruiz, 2021). A key downstream transcription factor of ER stress is the activating transcription factor 4 (ATF4) which mediates the expression of genes that allow cells to adapt cellular stress (Andrews et al., 2021). Indeed, the expression of ATF4 was significantly increased by glucose-restriction in A549, H1975 and H1299 cells (Figure 1F). Consistently, ATF4 was upregulated upon the treatment of thapsigargin which is a classical chemical inducer of ER stress (Figures 1G, H). Of note, a clear trend of co-expression of PCK2 and ATF4 was observed in A549 and H1975 cell lines (Figures 1F–H). To further determine the role of ATF4 in the induction of PCK2, we knocked down ATF4 through small interfering RNA (siRNA) and the expression of PCK2 was evaluated. As shown in Figures 1I, J, knockdown of ATF4 reduced both mRNA and protein levels of PCK2 in A549 and H1975 cells under glucose restriction conditions, indicating that the upregulation of PCK2 is dependent on ATF4. Then, we identified two putative ATF4 binding sites on the promoter of PCK2 through JASPAR database (Figure 1K). The ATF4 and PCK2 DNA direct interaction was experimentally demonstrated using ChIP assay (Figure 1L). Taken together, these data show that PCK2 is upregulated by glucose deprivation through ER stress-induced ATF4 expression in NSCLC cells.

### 2.2 Glucose deprivation enhances the glutamine fueling of the TCA cycle and gluconeogenesis

**FIGURE 2:**
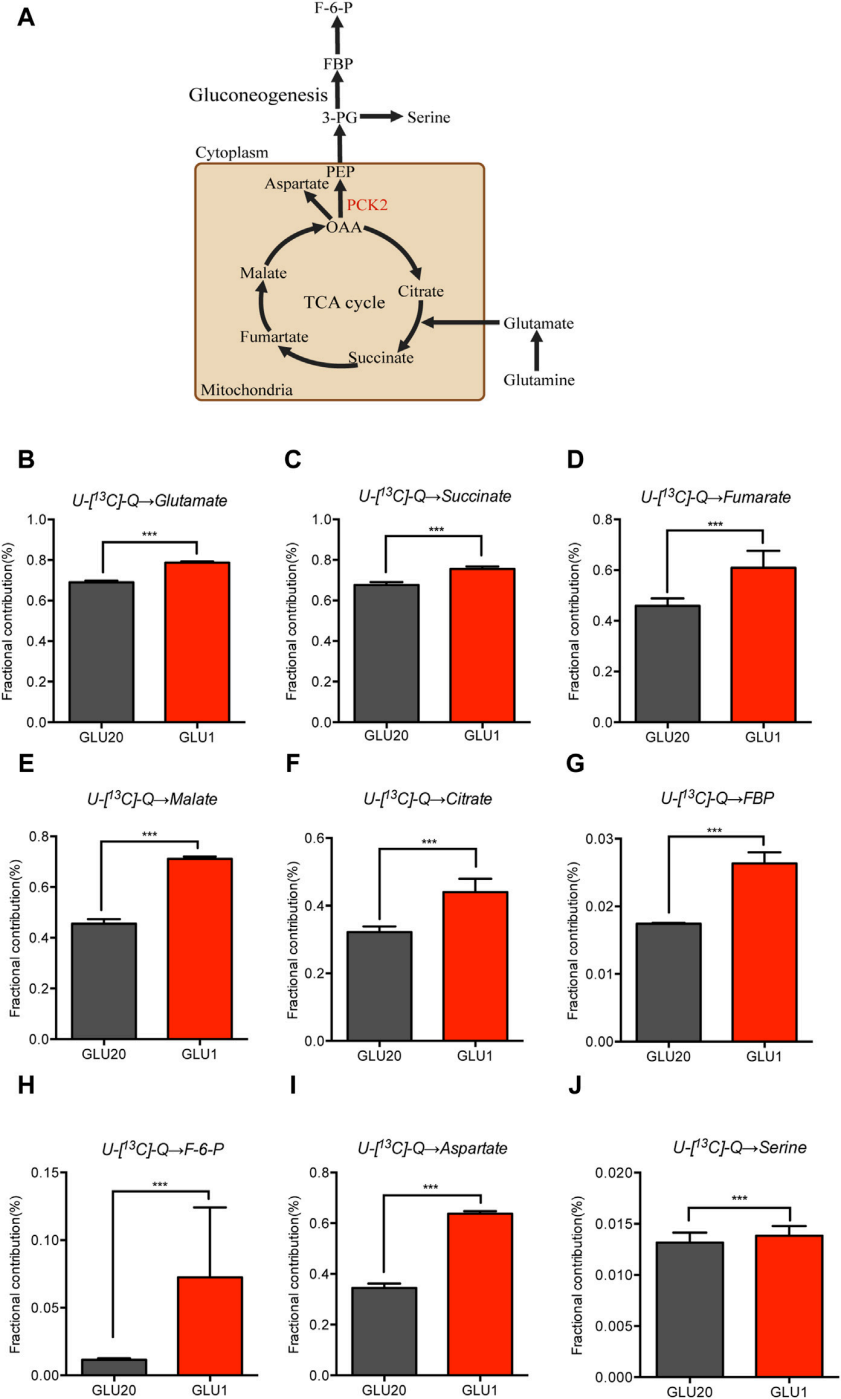
Glucose deprivation enhances the glutamine fueling of the TCA cycle and gluconeogenesis. **(A–J)** U-[13C]-glutamine-labelled glutamate **(B)**, TCA cycle metabolites **(C–F)**, gluconeogenesis metabolites **(G,H)**, Aspartate **(I)** and Serine **(J)** in A549 cells cultured in medium containing high (20 mM) and low (1 mM) concentrations of glucose for 12 h ***p < 0.001. (n = 4, mean ± SEM). U-[13C]-Q, U-[13C]-glutamine.

As enhanced glycolysis is essential for cancer cells to meet the demand for energy production, building blocks generation and redox homeostasis, how these cells maintain proliferation and energy supply under glucose restriction conditions remains poorly understood. Previous studies demonstrated that cancer cells increase oxidative phosphorylation to compensate for the reduced glycolysis, in which glutamine was used as an alternative substrate to supply the TCA cycle (Jin et al., 2023). To test if the contribution of glutamine to the TCA cycle is elevated by glucose deprivation in NSCLC cells, we employed stable isotope tracing metabolomics technology which allow precise quantification of metabolic fluxes of glutamine. As shown in Figures 2A–F, flux contribution of glutamine carbons to the TCA cycle was significantly increased by glucose deprivation as indicated by the enhanced ^13^C-labeled glutamate and TCA cycle metabolites including succinate, fumarate, malate and citrate. The TCA cycle links gluconeogenesis through the conversion of oxaloacetate into phosphoenolpyruvate by PCK2 in cytosol (Figure 2A). We then assessed the alteration of gluconeogenesis by quantifying the flux contribution of glutamine to metabolites belonging to the glycolysis/gluconeogenesis pathways. Importantly, we found an elevated ^13^C-labeled metabolites in the gluconeogenetic pathway, including fructose-1,6-bisphosphate (FBP) and fructose 6-phosphate (F-6-P) (Figures 2G, H). Aspartate, which is a key metabolite that supports nucleotide and asparagine synthesis in cancer cells, also increased its ^13^C-labelled part under glucose restriction conditions (Figure 2I). Moreover, an increased glutamine-dependent serine biosynthesis through gluconeogenesis was also observed after glucose deprivation (Figure 2J). The labeling ratios of gluconeogenetic metabolites were lower than that of malate, further indicating that these metabolites were the downstream intermediates of the TCA cycle. Collectively, these data demonstrate that NSCLC cells increase glutamine utilization under glucose restriction conditions, which in turn maintains TCA cycle operation and promote gluconeogenesis.

### 2.3 PCK2-mediated gluconeogenesis protects NSCLC cells against mitochondrial apoptosis under glucose-restriction conditions

**FIGURE 3:**
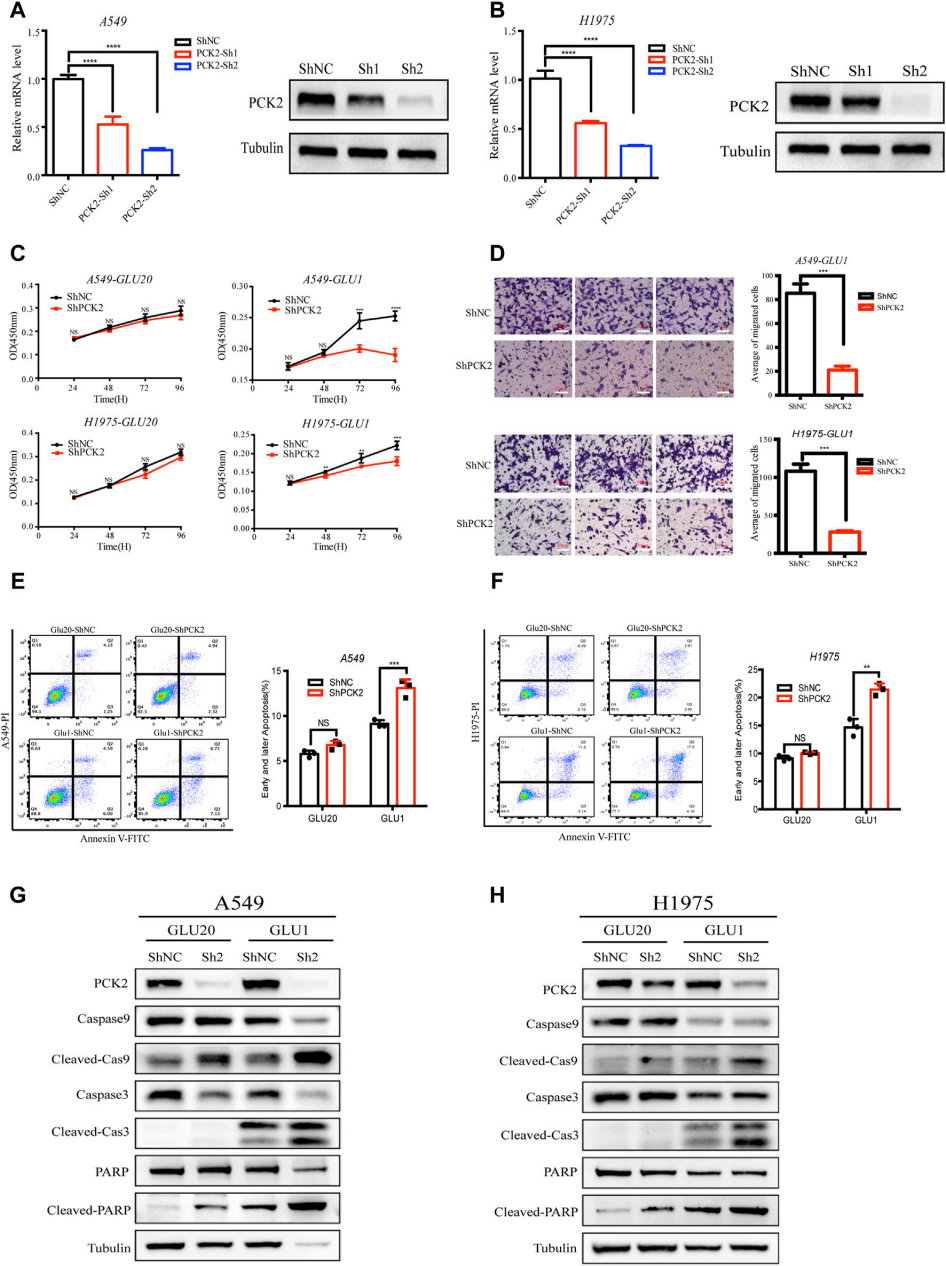
PCK2-mediated gluconeogenesis protects NSCLC cells against mitochondrial apoptosis under glucose-restriction conditions. **(A)** Expression of *PCK2* mRNA and protein in A549 transfected with control or PCK2 shRNA. ****p < 0.0001. Data are representative of three independent experiments (n = 3, mean ± SEM). **(B)** Expression of PCK2 mRNA and protein in H1975 cells transfected with control or PCK2 shRNA. Data are representative of three independent experiments. **(C)** Cell viability of A549 and H1975 cells transfected with control or PCK2 shRNA and cultured in medium containing high (20 mM) and low (1 mM) concentrations of glucose for 96 h **p < 0.01, ***p < 0.001, ****p < 0.0001. Data are representative of three independent experiments (n = 5, mean ± SEM). **(D)** Right: Representative images of transwell assays of A549 and H1975 cells transfected with control or PCK2 shRNA and cultured in medium containing 1 mM glucose for 48 h. Left: Statistics of cell migration of A549 and H1975 cells treated as in (Right). ***p < 0.001. Data are representative of three independent experiments (n = 3, mean ± SEM). **(E)** Right: Flow cytometry analysis of early and later apoptosis of A549 cells transfected with control or PCK2 shRNA and cultured in medium containing high (20 mM) and low (1 mM) concentrations of glucose for 48 h. Left: Statistics of early and later apoptosis of A549 cells treated as in (Right). ***p < 0.001; ns, no significant difference. Data are representative of three independent experiments (n = 3, mean ± SEM). **(F)** Right: Flow cytometry analysis of early and later apoptosis of H1975 cells transfected with control or PCK2 shRNA and cultured in medium containing high (20 mM) and low (1 mM) concentrations of glucose for 48 h. Left: Statistics of early and later apoptosis of A549 cells treated as in (Right). **p < 0.01; ns, no significant difference. Data are representative of three independent experiments (n = 3, mean ± SEM). **(G,H)** Expression of Caspase-9, Caspase-3, PARP and their cleaved forms in A549 **(G)** and H1975 **(H)** cells transfected with control or PCK2 shRNA and cultured in medium containing high (20 mM) and low (1 mM) concentrations of glucose for 48 h. Data are representative of three independent experiments.

Having shown that more glutamine contributes to the TCA cycle and gluconeogenesis in response to glucose deprivation, we next asked if this metabolic alteration affects the fate of cancer cells. As PCK2 is a rate-limiting enzyme of gluconeogenesis that converts oxaloacetate into phosphoenolpyruvate, we knocked down PCK2 by the small hairpin RNA (shRNA) to evaluate the role of PCK2-mediated gluconeogenesis in cell proliferation as well as cell death. The knockdown efficacy was confirmed at both the mRNA and protein levels (Figures 3A, B). While silencing of PCK2 did not affect the viability of A549 and H1975 cells cultured in regular medium, it significantly reduced the viability of these cells cultured in medium containing 1 mM glucose, indicating that PCK2-mediated gluconeogenesis is required for the optimal growth of NSCLC cell under glucose-restriction conditions (Figure 3C). In addition, PCK2 silencing reduced the migration abilities of A549 and H1975 cells, suggesting that PCK2-mediated gluconeogenesis is also essential for the invasion activity of NSCLC cells (Figure 3D). Numerous studies found that glucose restriction and sustained ER stress lead to the activation of pro-apoptotic signaling to drive apoptotic cell death (Chen and Cubillos-Ruiz, 2021; Fu et al., 2021). Indeed, we found that both early and later apoptosis rates of A549 and H1975 cells were slightly increased under glucose-restricted conditions (Figures 3E, F). Importantly, silencing of PCK2 significantly enhanced apoptosis rates of cancer cells cultured in glucose-restricted medium rather than in regular culture conditions (Figures 3E, F). These data demonstrate that PCK2-mediated gluconeogenesis is critical for NSCLC cells to acquire resistance to apoptosis under glucose restriction conditions.

Apoptosis is a process of cellular self-destruction catalyzed by many proteolytic enzymes, among which caspases play key roles in carrying out the cleavage of apoptosis-executive proteins (Boice and Bouchier-Hayes, 2020; Carneiro and El-Deiry, 2020). The activation of caspase three and caspase nine lead to the cleavage of Poly (ADP-ribose) polymerase (PARP) which is a hallmark of mitochondrial apoptosis (Carneiro and El-Deiry, 2020; Morana et al., 2022). To further investigate how PCK2-mediated gluconeogenesis inhibits apoptotic cell death of NSCLC cells, we tested the expressions of caspase 9, caspase 3, PARP and their cleaved forms in A549 and H1975 after PCK2 silencing. We found that the cleavages of caspase-9, caspase-3 and PARP were clearly induced in NSCLC cells cultured in medium containing l mM glucose but not in those cultured in regular medium (Figures 3G, H), confirming that glucose restriction trigger the activation of mitochondrial apoptotic signaling. Importantly, PCK2 silencing further increased the cleavages of caspase-9, caspase-3 and PARP (Figures 3G, H), indicating that PCK2-mediated gluconeogenesis is required to prevent the activation of mitochondrial pro-apoptotic signaling in NSCLC cells.

### 2.4 PCK2-mediated gluconeogenesis is required to reduce the burden of the TCA cycle and to rebalance redox equilibrium

**FIGURE 4:**
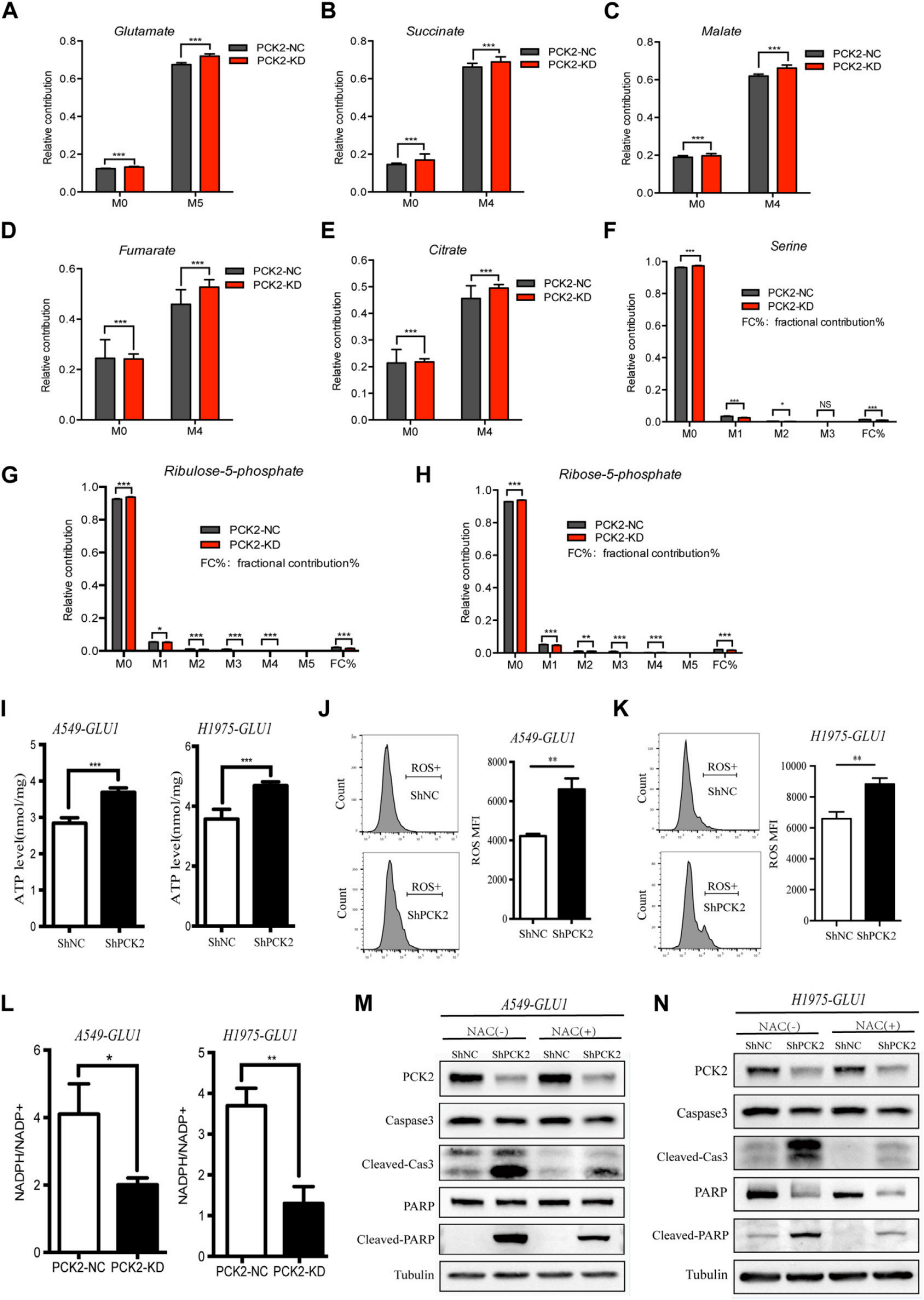
PCK2-mediated gluconeogenesis is required to reduce the burden of TCA cycle and to rebalance redox equilibrium. **(A–H)** U-[^13^C]-glutamine-labelled metabolites in A549 cells transfected with control or PCK2 shRNA and cultured in medium containing low (1 mM) concentrations of glucose for 12 h *p < 0.05, **p < 0.01, ***p < 0.001; ns, no significant difference. (n = 4, mean ± SEM). **(I)** Intracellular ATP levels of A549 and H1975 cells transfected with control or PCK2 shRNA and cultured in medium containing low (1 mM) concentrations of glucose for 48 h ***p < 0.001. (n = 3, mean ± SEM). **(J,K)** Flow cytometry analysis of mitochondrial ROS in A549 **(J)** and H1975 **(K)** cells transfected with control or PCK2 shRNA and culture in 1 mM glucose for 48 h **p < 0.01. Data are representative of three independent experiments (n = 3, mean ± SEM). **(L)** NADPH/NADP + ratio in A549 and H1975 cells transfected with control or PCK2 shRNA and cultured in medium containing 1 mM glucose for 48 h *p < 0.05, **p < 0.01. Data are representative of three independent experiments (n = 3, mean ± SEM). **(M,N)** Expression of PCK2 and Caspase-3, PARP and their cleaved forms in A549 **(M)** and H1975 **(N)** cells transfected with control or PCK2 shRNA, cultured in medium containing 1 mM glucose and 5 mM NAC for 48 h. NAC, N-acetyl cysteine. Data are representative of three independent experiments.

PCK2 mediates transport of oxaloacetate from the TCA cycle and decarboxylation to PEP, which has been recognized as an important metabolic adaptation for cancer cells in response to nutrition deprivations. However, little is known about how this metabolic alteration affects apoptotic caspases signaling. Since glucose restriction resulted in the accumulation of glutamine-derived TCA cycle metabolites (Figures 2C–F), we hypothesized that PCK2-mediated gluconeogenesis is required to remove excess intermediates of the TCA cycle and in turn alleviate mitochondria-associated cellular dysfunction which is a key driver of apoptotic cell death. In support of this hypothesis, we found that ^13^C-glutamine labeled glutamate and TCA cycle metabolites, including citrate, succinate, fumarate and malate, were increased by PCK2 silencing in A549 cells cultured in medium containing low (1 mM) concentrations of glucose. (Figures 4A–E). Conversely, ^13^C-glutamine derived serine as well as metabolites involved in pentose phosphate pathway including ribulose-5-phosphate and ribose-5-phosphate were reduced by PCK2 silencing (Figures 4F–H).

The accumulation of TCA cycle metabolites is associated with increased oxidative phosphorylation and reactive oxygen species (ROS) production (Chakrabarty and Chandel, 2021). As PCK2-mediated gluconeogenesis is required to reduce the burden of the TCA cycle in NSCLC cells under glucose-restriction conditions, we tested if PCK2 silencing affects ATP generation and mitochondrial ROS(mtROS) production in this setting. As shown in Figures 4I–K, the levels of both ATP and mtROS were elevated by PCK2 silencing in A549 and H1975 cells. The increased ATP level after PCK2 silencing is likely due to the enhanced oxidative phosphorylation as ATP generation through glycolysis has been impaired by glucose restriction in this setting. The mitochondrial respiratory chain is the main generator of mtROS which is considered as byproduct of oxidative phosphorylation during ATP synthesis. The increased level of mtROS after PCK2 silencing suggests that either more mtROS was generated due to enhanced OXPHOS, as indicated above, or less mtROS was detoxified. In addition, or less gluconeogenesis is required to reduce the production of mtROS maintain redox balance in NSCLC cells upon glucose deprivation (Figures 4J, K). The later was supported by the findings that PCK2 silencing decreased glutamine-derived metabolites within the pentose phosphate pathway (Figures 4G, H), through which nicotinamide adenine dinucleotide phosphate (NADPH) was generated for ROS detoxification (Hayes et al., 2020). To further confirm this, we measured the NADPH/NADP ratio in NSCLC cells after silencing of PCK2. We found that NADPH/NADP ratio was reduced by PCK2 silencing in both A549 and H1975 cells, showing that cellular redox balance was disrupted (Figure 4L).

As mtROS is an important inducer of apoptotic cell death, we next tested if the protective role of PCK2-mediated gluconeogenesis in NSCLC cells upon glucose deprivation is through inhibiting mtROS production. To this end, A549 and H1975 cells were incubated in glucose starvation medium containing N-acetyl cysteine (NAC), which is a direct oxidant scavenger of ROS, before the evaluation of the activation of mitochondrial pro-apoptotic signaling. As shown in Figures 4M, N, the increased cleavages of caspase-3 and PARP by PCK2 silencing under glucose restriction conditions were largely attenuated by NAC, conforming that mtROS is the key mediator of mitochondrial apoptosis in this setting. Taken together, these data showed that PCK2-mediated gluconeogenesis is required to reduce the accumulation of TCA cycle metabolites and the level of oxidative phosphorylation, which in turn decreases mtROS production and apoptotic cell death of NSCLC cells upon glucose restriction.

### 2.5 PCK2 promotes lung tumorigenesis and metastasis *in vivo*

**FIGURE 5:**
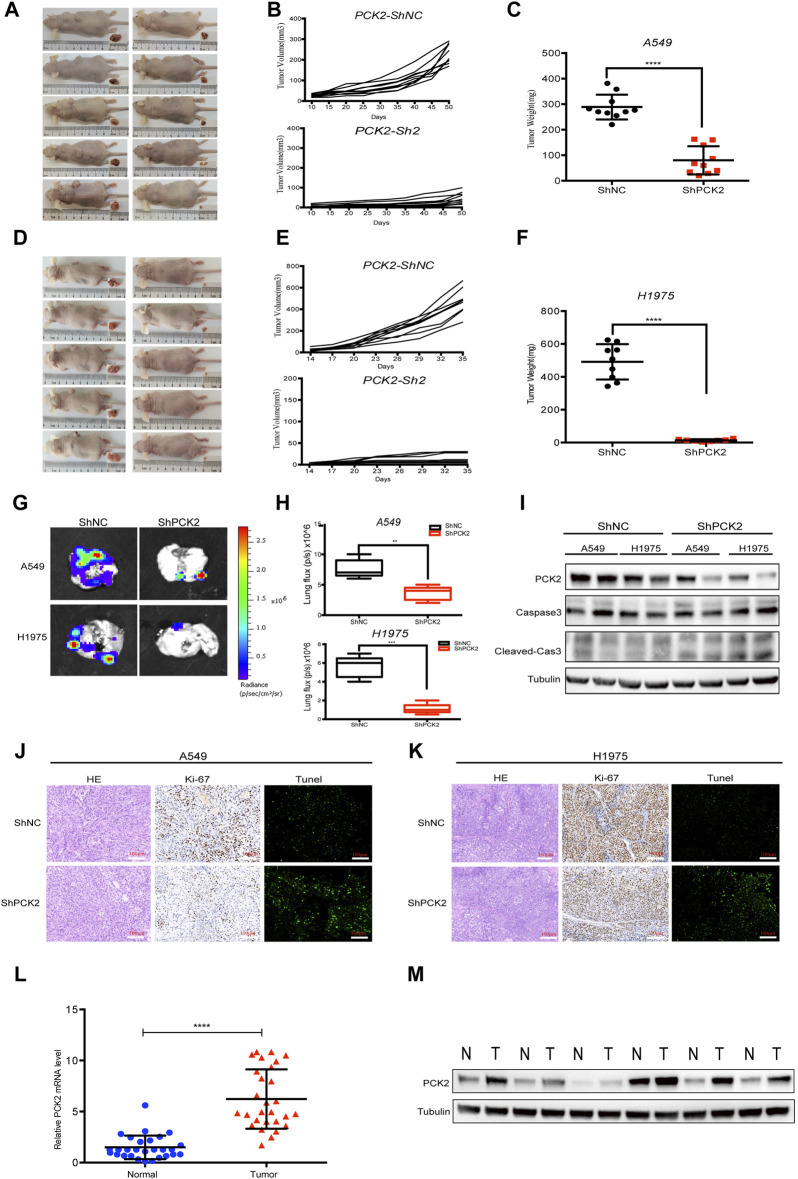
PCK2 promotes lung tumorigenesis and metastasis *in vivo*. **(A–C)** Representative photographs of tumors, tumor volume and weight in xenografts mice models after 50 days subcutaneously injection of A549 cells transfected with control or PCK2 shRNA. ****p < 0.0001. (n = 10 mice, mean ± SEM). **(D–F)** Representative photographs of tumors, tumor volume and weight in xenografts mice models after 35 days subcutaneously injection of H1975 cells transfected with control or PCK2 shRNA. ****p < 0.0001. (n = 10 mice, mean ± SEM). **(G,H)** Representative photographs of lung metastasis of mice after 45 days injection of A549 and H1975 cells transfected with control or PCK2 shRNA. **p < 0.01, ***p < 0.001; ns, no significant difference. (n = 5 mice, mean ± SEM). **(I)** Expression of PCK2, Caspase-3, cleaved-Caspase-3 in subcutaneously implanted tumor tissues of A549 and H1975 cells transfected with control or PCK2 shRNA. Data are representative of three independent experiments with ten mice tested in each group. **(J,K)** Representative images of HE, Ki67 and Tunel staining of the tumor sections in subcutaneously implanted tumors of A549 **(J)** and H1975 **(K)** cells transfected with control or PCK2 shRNA. Images are obtained from ten mice in each group. **(L)** Quantification of PCK2 mRNA expression from tumor samples and matched adjacent normal lung tissues of NSCLC patients (n = 29). *p < 0.0001. **(M)** Immunoblot for PCK2 on six individual samples of normal and tumor tissue samples from NSCLC patients. Tubulin was used as a control for protein loading. Data are representative of three independent experiments.

**FIGURE 6:**
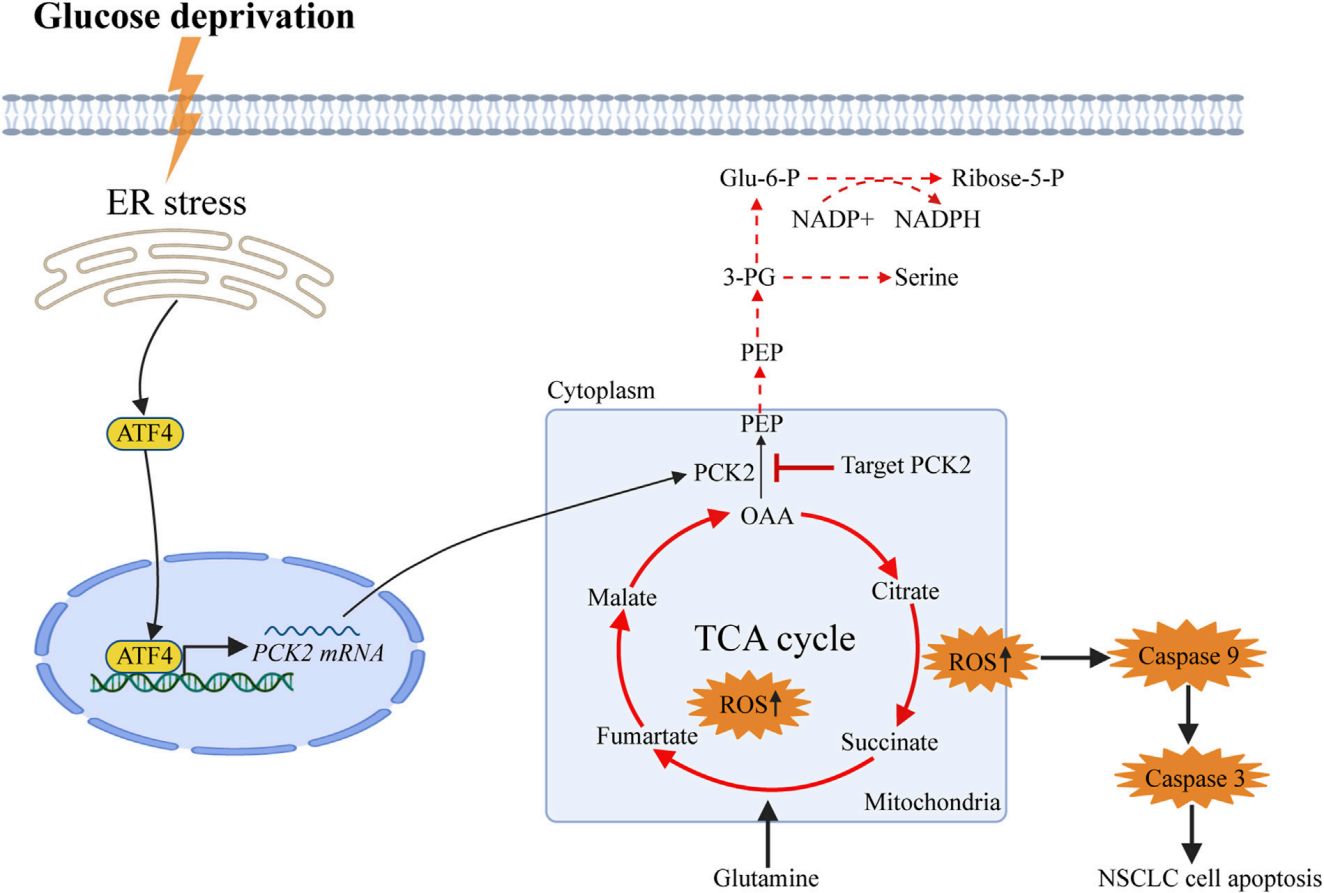
Working model of PCK2-mediated metabolism promotes lung tumorigenesis. Phosphoenolpyruvate Carboxykinase 2 (PCK2) is upregulated in dependent of endoplasmic reticulum stress-induced expression of activating transcription factor 4 (ATF4) upon glucose deprivation in NSCLC cells. PCK2-mediated metabolism is required to decrease the burden of the TCA cycles and oxidative phosphorylation as well as the production of mitochondrial reactive oxygen species, in turn reduce the activation of Caspase9-Caspase3-PARP mitochondrial apoptotic pathway.

To assess the impact of PCK2-mediated metabolism on tumor growth *in vivo*, A549 and H1975 cells expressing either control or PCK2-specific shRNAs were injected into the flanks of nude mice, and tumor growth was assessed every 2 days. We found that tumor volumes and weights of both A549-shPCK2 and H1975-shPCK2 were significantly decreased compared to those of control cells, indicating the critical role of PCK2 in the growth of NSCLC cells *in vivo* (Figures 5A–F). We next evaluated if PCK2-mediated gluconeogenesis affects metastatic capacity of NSCLC cells. To this end, we intravenously injected luciferase-expressing A549 and H1975 cells transfected with either control or PCK2-specific shRNAs in mice and lung metastasis was evaluated after 45 days. Strikingly, both A549-shPCK2 and H1975-shPCK2 dramatically reduced their metastatic capacity compared to control cells as indicated by the significantly reduced percentage of tumor area within the lung (Figures 5G, H). Importantly, the expression of cleaved caspase-3 and the number of TUNEL positive cells were increased in both A549-shPCK2 and H1975-shPCK2 tumor tissues (Figures 5I–K), confirming that loss of PCK2 promotes apoptosis in NSCLC cells *in vivo* in the low glucose. Moreover, there were less Ki67-positive cells within tumor tissue upon PCK2 knocking down, suggesting a reduced proliferative capacity of these cells (Figures 5J, K). Taken together, our data show that PCK2-mediated gluconeogenesis promotes tumorigenesis of NSCLC *in vivo* by protecting lung cancer cells against apoptosis. We also assessed PCK2 mRNA and protein levels in tumor samples isolated from human NSCLC patients. Real-time PCR revealed a significant increase in PCK2 mRNA expression levels in lung tumors compared with adjacent normal tissue (Figure 5L). Examples of PCK2 protein expression in normal and tumor tissue from the lungs of six NSCLC patients are shown in Figure 5M. These data indicate that PCK2 expression is enriched in the tumor tissues of NSCLC. A working model of PCK2-mediated metabolism promotes lung tumorigenesis has been shown in Figure 6.

## 3 Discussion

Solid tumors frequently encounter nutrient deprivation during growth due to insufficient blood supply (Martínez-Reyes and Chandel, 2021). Since cancer cells prefer the process of glycolysis as the source of ATP generation and macromolecular synthesis (Vander Heiden et al., 2009), it is unclear how these cells maintain viability and proliferation in the microenvironment with limited availability of glucose. In this study, we demonstrate that PCK2 is upregulated in NSCLC cells in response to glucose deprivation through ER stress-induced ATF4 expression. PCK2-mediated gluconeogenesis protects NSCLC cells against mitochondrial apoptosis by reducing the burden of the TCA cycle and restoring cellular redox balance. Importantly, silencing of PCK2 inhibits the growth of NSCLC cells both *in vitro* and *in vivo* in the low glucose condition, suggesting that PCK2 is a potential therapeutic target for NSCLC. To date, there has been little focus on PCK2 in the context of cancer. Elevated expression of the upstream gluconeogenesis enzyme PCK2 has been noted in many tumor types include colon cancer, NSCLC and hepatocellular carcinoma. In this light, targeting PCK2 may be an effective strategy for certain cancer subtypes. However, recently, several studies had suggested that PCK2 overexpression could suppress the progression of renal cell carcinoma and melanoma (Luo et al., 2017; Xiong et al., 2020). The tumor-promoting or tumor-suppressive roles of PCK2 in different studies depend on the cancer type and context. Thus, the diversity of PCK2 expression in different cancer models make it a challenging therapeutic target and it is required to fully investigate the underlying molecular mechanisms of PCK2-mediated tumor development in different tissues and disease stages (Yu et al., 2023). Additionally, to identify specific tumor types that can be effectively treated by targeting PCK2, it is imperative to develop the biomarkers to predict the response of tumor cells against PCK2 inhibitors. It is worth noting that PCK2-mediated metabolic downstream pathways play an important role to maintain physiology functions of liver and insulin-producing β-cells (Stark et al., 2009; Stark et al., 2014), whether targeting PCK2 could affect these normal organs and tissues is still undermined and need to be addressed in future studies.

Gluconeogenesis is required for cancer cells to generate glycolytic intermediates in adapting to a glucose-deprived tumor microenvironment (Wang and Dong, 2019). A key step in gluconeogenesis is the conversion of oxaloacetate to phosphoenolpyruvate, which is catalyzed by PCK1 in the cytoplasm or by PCK2 in the mitochondrial (Grasmann et al., 2019). While PCK1 is the dominant form of PCK in the liver, the active and functional form of PCK in cancer cells is dependent on tumor types (Bian et al., 2022). PCK1 is upregulated and required for optimal growth of human pancreatic cancer cells, hepatocellular carcinoma cells and melanoma cells (Xu et al., 2020). Conversely, PCK2 was shown to be critical for the proliferation of lung cancer cells, kidney renal clear cancer cells and breast cancer cells (Chen et al., 2023; Hsu et al., 2023). In line with previous studies (Leithner et al., 2015), we found that PCK2 rather than PCK1 is upregulated in NSCLC cells upon glucose deprivation. Importantly, we uncovered that glucose restriction-induced ER stress and its downstream ATF4 upregulation are the key drivers of PCK2 expression in NSCLC cells. Glucose deprivation induces ER stress in cancer cells via multiple ways. The compromised glycolysis impairs the production of uridine diphosphate-N-acetylglucosamine (UDP-GlcNAc) which is required for N-linked glycosylation and protein folding in the ER (Xiang et al., 2021). In addition, glucose restriction leads to defected calcium flux in the ER (Li et al., 2019). Although it is well established that ER stress leads to a decrease in global protein translation, it also upregulates specific proteins to reduce the burden of unfolded proteins via the transcription factor ATF4 (Balsa et al., 2019). In this study, we confirmed that glucose deprivation leads to ER stress and ATF4 upregulation in NSCLC cells. Of note, we found that the induction of PCK2 is dependent on ATF4 expression, revealing a novel mechanism of how PCK2 is induced under nutrient deprivation conditions in NSCLC cells. Since PCK2-mediated metabolism is critical for the survival of NSCLC cells, targeting ER stress-ATF4 signaling pathway could be a therapeutic approach for the treatment of NSCLC.

Apoptotic cell death is an important form of regulated cell death which plays a pivotal role in cancer development (Kashyap et al., 2021). Evasion of apoptosis is not only a common characteristic of many types of cancer cells, but also is a crucial ability for these cells to maintain optimal growth under nutrient-deprived tumor microenvironment (Shahar and Larisch, 2020). Of note, mitochondria are critical to trigger apoptotic signaling. Unlike extrinsic pathway of apoptosis, mitochondrial apoptosis is induced by diverse cellular stresses such as growth-factor deprivation, DNA damage or glucose deprivation (Caro-Maldonado et al., 2010; Luo et al., 2020). In this study, we confirmed that glucose deprivation promotes mitochondrial apoptosis in NSCLC cells. Intriguingly, the extent of this form of apoptosis was significantly increased by PCK2 silencing in NSCLC cells under glucose-limited conditions but not in cells grown in regular culture medium, indicating that PCK2 is essential for NSCLC cells to acquire resistance to mitochondrial apoptosis upon nutrient deprivation. We further revealed that PCK2-mediated gluconeogenesis removes abundance TCA cycle metabolites and restores redox balance in NSCLC cells upon glucose deprivation, showing the fine-tuning metabolic switches in cancer cells in response to nutrient-deprived tumor microenvironment. Given the induction of apoptosis has been recognized as a promising approach for the treatment of cancers (Mohammad et al., 2015), the current study provides a new therapeutic target to inhibit tumor growth of NSCLC by promoting apoptosis.

In summary, our study demonstrates that PCK2-mediated gluconeogenesis is critical for NSCLC cells to maintain survival under glucose-limited conditions. Targeting PCK2 could be used as a potential therapeutic strategy for the treatment of non-small-cell lung cancer.

## 4 Materials and methods

### 4.1 Cell lines and culture

The Human NSCLC cell lines (A549, H1299, and H1975) were obtained from the American Type Culture Collection. Cells were cultured in DMEM (A549 cells) or RPMI 1640 (H1299 and H1975) medium supplemented with 10% fetal calf serum (FCS) and antibiotics (100 U/mL penicillin and 0.1 mg/mL streptomycin) at 37°C in a humidified incubator containing 5% CO2. For high and low glucose experiments, glucose- and glutamine-free DMEM (Gibco) were supplemented with 2 mM glutamine and 1/20 mM (low/high) glucose, 10% FCS and antibiotics. All cell lines were tested for *mycoplasma* contamination before use and validated by short tandem repeat profiling.

### 4.2 Isotope-tracing experiments

Metabolic tracing analysis of U-[^13^C]-glutamine (Cat#CLM-1822-H, Cambridge Isotope Laboratories) in A549 cells were determined by GC–MS or LC–MS. Briefly, 1 × 106 A549 cells were seeded in 3.5 cm dishes in regular medium (DMEM supplemented with 10% FCS and antibiotics) for 18 h. Cells were then washed twice with phosphate buffered saline (PBS) and incubated in medium containing 1 mM or 20 mM glucose with 2 mM glutamine for 24 h. After 3 times wishing by PBS, cells were incubated in medium containing 1 mM or 20 mM glucose as well as 2 mM U-[^13^C]-glutamine for 12 h. Cells were then collected and processed as described for intracellular metabolites. Metabolites were quantitated by Metabo-Profile Biotechnology (Shanghai, China). To determine ^13^C-labelling, mass information for known fragments of labelled metabolites was retrieved. These fragments contained either the whole or partial carbon skeleton of the metabolite. For each fragment, the retrieved data consisted of mass intensities for the lightest isotopomer (without any heavy isotopes, M + 0) and isotopomers with increasing unit mass (up to M + 6) relative to M0. These mass distributions were normalized by dividing by the sum of M0 to M6 and corrected for the natural abundance of heavy isotopes, using matrix-based probabilistic methods as described previously, (Lee et al., 1991), and implemented in Microsoft Excel. ^13^C-labelling data are expressed as fractional abundance of each isotopolog of a measured metabolite pool or relevant enrichment of each metabolite. Fractional contribution (%) is determined by the formula (FC% = (M1*1 + M2*2+…+Mn*n)/n) as described in the previous literature. (Buescher et al., 2015).

### 4.3 RNA-sequence

Total RNA was extracted using the TRIzol reagent (Invitrogen, CA, USA) according to the manufacturer’s protocol. RNA purity and quantification were evaluated using the NanoDrop 2000 spectrophotometer (Thermo Scientific, USA). RNA integrity was assessed using the Agilent 2,100 Bioanalyzer (Agilent Technologies, Santa Clara, CA, USA). The libraries were constructed using VAHTS universal V6 RNA-seq Library Prep Kit according to the manufacturer’s instructions. The transcriptome sequencing and analysis were conducted by OE Biotech Co., Ltd. (Shanghai, China). The libraries were sequenced on an Illumina HiSeq X Ten platform and 150 bp paired-end reads were generated. Raw reads for each sample were generated. Raw data (raw reads) of fastq format were firstly processed using Trimmomatic (Bolger et al., 2014) and the low quality reads were removed to obtain the clean reads. The clean reads for each sample were then retained for subsequent analyses. The clean reads were mapped to the human genome (GRCh38) using HISAT2 (Kim et al., 2015). FPKM (Roberts et al., 2011) of each gene was calculated using Cufflinks (Trapnell et al., 2010), and the read counts of each gene were obtained by HTSeq-count (Anders et al., 2015). Differential expression analysis was performed using the DESeq (2012) R package. *p*-value <0.05 and foldchange >2 or foldchange <0.5 was set as the threshold for significantly differential expression. Hierarchical cluster analysis of differentially expressed genes (DEGs) was performed to demonstrate the expression pattern of genes in different groups and samples. GO enrichment and KEGG (Kanehisa et al., 2008) pathway enrichment analysis of DEGs were performed respectively using R based on the hypergeometric distribution.

### 4.4 Quantitative real-time PCR

Total RNA was isolated from NSCLC cell lines using RNeasy Plus Mini Kit (Cat#74134, QIAGEN), and 2,000 ng of RNA was used for the cDNA synthesis using a PCR cDNA kit according to manufacturer’s protocol (Cat#K1622, Thermofisher). Quantitative real-time PCR was performed using the PowerUp SYBR Green Master Mix (Thermo Fisher Scientific) on a QuantStudio 6 Flex System (Applied Biosystems). All samples were measured in triplicate and the mean value was used. The gene expression levels were calculated according to the 2^−ΔΔCT^ method and normalized to 18S rRNA. The primers sequence used for gene expression analysis in this study were listed in Supplementary Table S1.

### 4.5 Western blotting

After different treatments and stimulation, cells were lysed in RIPA buffer containing Complete Mini EDTA-Free protease inhibitor cocktail and phosphatase inhibitor cocktail (Roche). Protein concentrations of cell lysate were measured by Pierce BCA protein assay kit (Thermo Fisher Scientific) and equal amounts of total protein from different samples were used. Cell lysate was then boiled in SDS sample buffer for 5 min at 95°C–100°C, separated by SDS–PAGE and transferred to PVDF membranes (Bio-Rad). The membrane was then blocked in TBST plus 5% non-fat dry milk (Bio-Rad) for 1hincubated with primary antibodies at 4°C overnight. After three times washing with TBST, the membranes were incubated with HRP-linked secondary antibodies for 1 h at room temperature (RT) and visualized with Clarity Western ECL substrates (Thermo Fisher Scientific). Bands of interest were developed by using an autoradiographic film. The antibodies used for the immunoblotting studies are listed in Supplementary Table S2.

### 4.6 Cell proliferation, migration and invasion

To test cell proliferation rate, NSCLC cells were seeded in a 96-well plates at the density of 5 × 10^4^/well in 100 μL complete medium for 18–24 h. Cells were then washed twice with PBS and incubated in the medium containing high glucose or low concentrations of glucose. Cell viability was assessed at 24, 48, 72 and 96 h using a Cell Counting K-8 kit-8 (Dojindo, Japan) according to the manufacturer’s instructions.

To test migration and invasion of lung cancer cells, 3 × 10^5^/well NSCLC cells were seeded on the top chamber of the transwell plate (Corning) in serum-free mediun containing high glucose or low concentrations of glucose medium, and medium supplemented with 10% FCS was added to the bottom chamber. After 48 h of incubation at 37°C, the chambers were fixed with methanol for 30 min, followed by staining with crystal violet (C0121, Beyotime, China) for another 30 min. The average number of migrated cells per representative field was calculated by ImageJ software.

### 4.7 Gene silencing by siRNA or shRNA transfection

Small interfering RNAs (SiRNAs) were used to induce transient knockdown of ATF4 and PCK2 in NSCLC cells. All siRNA duplexes were purchased from GenePharma (GenePharma, Shanghai, China). Briefly, the human NSCLC cell lines were seeded in the six-well plates at a density of 3 × 10^5^ cells/well for 18–24 h in regular medium. The cells were then washed twice with PBS and cultured in the medium containing high glucose or low concentrations of glucose. Before transfection, diluted siRNA was mixed with diluted Lipofectamine™ RNAiMAX in OptiMEM medium (Cat#13778150, Invitrogen, USA) and was incubated for 15 min at room temperature. The mixtures were added to the culture medium with the final concentration of SiRNA at 50 nM. Cells were transfected with different siRNA for 48 h before further experimentation.

PCK2 shRNAs and nonsense control shRNA were inserted into the plasmid vector GV248 and lentiviruses were constructed, which were purchased from Shanghai GeneChem Co., Ltd. The name of the empty plasmid was hU6-MCS-Ubiquitin-EGFP-IRES-puromycin. The scrambled or shPCK2 constructs were transfected with 293T cells with the pack-aging plasmids PMD2G and psPAX2 using Lipofectamine 2000 (Invitrogen Life Technologies) for 48 h according to the manufacturer’s instructions. Later, the retrovirus titer in the culture supernatant was collected. 1.5 mL of viral supernatant and 1.5 mL of fresh medium were then added to in the six-well plate containing A549 and H1975 cell lines in the presence of hexadimethrine bromide (polybrene), respectively. After 48 h, positively infected cells were selected using puromycin at 2 μg/mL. All siRNA or shRNA sequences are shown in Supplementary Table S3.

### 4.8 Flow cytometric analysis of cell apoptosis

A549 and H1975 cell lines were seeded in the six-well plates at a density of 5 × 10^5^ cells/well for 18–24 h in regular medium. After twice washing with PBS, cells were cultured in the medium containing high or low concentrations of glucose for 48 h. Cells were then harvested and stained with Annexin V-FITC and Propidium iodide (PI) (E-CK-A217, Elabscience, China) for 20 min at room temperature in the dark. The percentage of apoptotic cells for each sample was immediately analyzed by flow cytometer (CytoFlex, BeckmanCoulter).

### 4.9 Detection of mitochondrial ROS

To measure mitochondrial reactive oxygen species, cells were seeded in the 6-well paltes for 18 h in regular medium, followed by incubating in the medium containing low concentrations of glucose (1 mM) for 48 h. Cells were then collected and incubated with 5 μM of MitoSox (M36008, Invitrogen, USA) for 30 min at 37°C. After washing with warm PBS, cells were resuspended in PBS and analyzed by flow cytometry (CytoFlex, BeckmanCoulter).

### 4.10 Determination of ATP and NADPH/NADP + ratio

Intracellular ATP levels were determined using an ATP assay kit (S0026B, Beyotime, China) according to the manufacturer’s instructions. Briefly, NSCLC cells were plated overnight and cultured in the medium containing low concentrations (1 mM) of glucose for 48 h. The cells were then washed with cold PBS and lyzed in 200 μL ATP lysis buffer. The lysate was centrifuged at 12,000 g for 5 min, and the supernatants were used to analyze with the ATP level using ATP assay kit.

The NADPH/NADP + ratio was measured using NADP+/NADPH Assay Kit with WST-8 (S0179, Beyotime, China) according to the manufacturer’s instructions. Briefly, NSCLC cells were plated overnight and then cultured in the medium containing low concentrations (1 mM) of glucose for 48 h. The cells were then collected and lysed by three freeze-thaw cycles. The lysates were used to determine NADPH and NADP + respectively. The NADPH/NADP + ratio was calculated by the following formula: (NADPH)/(NADP_total_–NADPH).

### 4.11 Mouse strains

Sex- and age-matched C57 BALB/c nude mice (Vital River Laboratory Animal Technology Co. Ltd., Beijing, China) aged 6–8 weeks were used in this study. The mice were bred and maintained under specific pathogen-free conditions with *ad libitum* access to food and water. All the animal experiments were approved by the approval of the animal care and use of committee of Tongji University (Approval number: TJBB03723107).

### 4.12 Subcutaneous growth of xenotransplants and lung metastasis in nude mice

To evaluate the growth of NSCLC cells *in vivo*, A549 and H1975 cells expressing either ShNC or ShPCK2 (5 × 10^6^) suspended in 200 μL PBS were injected subcutaneously into the right flanks of athymic nude mice (age 6–8 weeks). Tumor length (L) and width (W) was measured every 2 days with a digital caliper and tumor volume (V) was calculated (V = L × W^2^/2). At the end of the experiments, mice were sacrificed, and tumors were dissected out and *ex vivo* weighted. Tumor tissues were collected for further experimentation.

To evaluate lung metastasis of NSCLC cells, 5 × 10^6^ luciferase-expressing A549 and H1975 ShNC or ShPCK2 cells suspended in 200 μL PBS were injected into the tail vein of the athymic nude mice (age 6–8 weeks). After 45 days, the mice were anesthetized with phenobarbital sodium and 150 mg/kg D-luciferin (Cat#D9390, Solarbio, China) was injected intraperitoneally. The mice were then scanned and monitored by bioluminescence imaging using the IVIS system. Finally, all nude mice were sacrificed, and the lungs were dissected out and photographed.

### 4.13 Immunohistochemistry and TUNEL assay

Tumor tissues for immunohistochemistry were fixed with neutral formalin and embedded in paraffin. Tumor sections were stained immunohistochemically with anti-Ki67 antibody (Cat#ab16667, Abcam, United Kingdom) as well as hematoxylin. The staining was visualized by adding 3, 3′ -Diaminobenzidine (DAB) (Cat#8059, Cell Signaling Technology, USA).

TUNEL assay was performed on tumor sections by using TUNEL assay kit (Cat#25869, Cell Signaling Technology, USA) following the manufacturer’s instructions. Briefly, tumor sections were deparaffinized with xylene and rehydrated gradually using ethanol. The sections were then permeabilized with Proteinase K and labeled with Terminal Deoxynucleotidyl Transferase, Recombinant, (rTdT) enzyme. Fluorescent detection of apoptotic cells was achieved by using fluorescence microscopy (NIKON ECLIPSE TI-SR) and NIKON DS-U3 Imaging System.

### 4.14 Chromatin immunoprecipitation (CHIP)-PCR

Putative binding sites of ATF4 on PCK2 promoter were predicted using online JASPAR database (https://jaspar.elixir.no/). A549 cells were subjected to ChIP assay to verify the potential protein-DNA interaction using a commercial kit (P2080S; Beyotime, Shanghai, China) following the manufacturer’s protocol. Briefly, cells were firstly treated with 1% formaldehyde for 10 min to cross link proteins and genomic DNA, then sonicated on ice to break down DNA into 400–800 base pairs. 1% of the lysed samples were taken as the input. Then, samples were pretreated with protein A/G magnetic beads to block non-specific bindings. Each sample was divided into two equal parts and incubated with anti-ATF4 (D4B8, Cell Signaling Technology) and anti-IgG (30000-0-AP; Proteintech, Wuhan, China) antibodies at 4°C overnight, respectively. Subsequently, samples were added with protein A/G magnetic beads, rotated at 4°C for 1 h and washed with the corresponding buffers. A small part of samples was subjected to Western blot assay to ensure immunoprecipitation efficiency. The rest samples were purified and subjected to quantitative PCR. Two primers were designed based on the putative binding sites. Primer 1: forward: 5’-TTC​CTA​GCT​TGT​TTG​CCA​CCT​A-3′, reverse: 5′-CCA​GCC​GCA​CAT​GAT​GTA​ACT​T-3’; primer 2: forward: 5′- ACA​CAA​AAG​TTG​GCT​AAG​CTG​C-3′, reverse: 5′- GGA​ACC​ATC​TCC​TCA​GTC​TGT-3’.

### 4.15 Clinical NSCLC specimens

Tumour and adjacent normal tissues were freshly collected during the surgery. All patients were pathologically and clinically diagnosed as NSCLC. Three distinct samples of lung tumor tissue and adjacent normal lung from the resection margin were taken, flash-frozen in liquid nitrogen, and stored at −80°C until further analysis. Only NSCLC tumor samples comprising at least 90% of tumor tissue were analyzed. Each tissue sample weighing approximately 50 mg were homogenized in 500 mL lysis buffer (cat#78510, thermo scientific) using a tissuelyser (JXFSTPRP-32L, Shanghai Jingxin) to generate tissue lysates for real-time PCR and Western blot. Patients’ consent and approval from the Ethics Committee of the East Affiliated Hospital of Tongji University were obtained before using these tissue materials for research purposes (Grant number:2023037).

### 4.16 Statistical analysis

All the data were presented as means ± standard error of the mean (S.E.M). Analyses were performed by using GraphPad Prism Software 6.0 (San Diego, CA, USA). Student t-test were used for two groups, and One-way ANOVA followed by Tukey’s test was used to compare more than two groups. Statistical significance is represented in figures as follows: *p < 0.05, **p < 0.01, ***p < 0.001 and ****p < 0.0001.

## Data Availability

The raw RNA sequence data reported in this paper have been deposited in the Sequence Read Archive Database in the NCBI (Accession number: PRJNA1111007). Further inquiries can be directed to the corresponding authors.
