# Supplementary material for: Phosphoenolpyruvate carboxykinase 2-mediated metabolism promotes lung tumorigenesis by inhibiting mitochondrial-associated apoptotic cell death
Source: Front Pharmacol. 2024 Aug 9;15:1434988. doi: 10.3389/fphar.2024.1434988 (PMC11347759; doi:10.3389/fphar.2024.1434988)
Supplement: Supplementary file 1 [file Table1.DOCX]

| Supplementary Table S1. Sequence of relative primers. | | |
| --- | --- | --- |
| Primers | Forward sequences(5’-3’) | Reverse sequence(5’-3’) |
| PCK2 | TGCCAGGCTGGAAAGTGGAGTGT | GCAACCCCAAAGAAGCCGTTCTCA |
| ATF4 | CTCCGGGACAGATTGGATGTT | GGCTGCTTATTAGTCTCCTGGAC |
| 18s RNA | GTAACCCGTTGAACCCCATT | CCATCCAATCGGTAGTAGCG |
