# Supplementary material for: Phosphoenolpyruvate carboxykinase 2-mediated metabolism promotes lung tumorigenesis by inhibiting mitochondrial-associated apoptotic cell death
Source: Front Pharmacol. 2024 Aug 9;15:1434988. doi: 10.3389/fphar.2024.1434988 (PMC11347759; doi:10.3389/fphar.2024.1434988)
Supplement: Supplementary file 2 [file Table2.DOCX]

| Supplementary Table 2. Antibody information used in this study. | | | |
| --- | --- | --- | --- |
| Antibody | Catalog Number | | Company |
| PCK2 | D3E11 | Cell Signaling Technology | |
| ATF4 | 11815 | Cell Signaling Technology | |
| GAPDH | 5174 | Cell Signaling Technology | |
| Tubulin | 66031 | Proteintech | |
| Caspase9 | 9504 | Cell Signaling Technology | |
| Cleaved-caspase9 | 9509 | Cell Signaling Technology | |
| Caspase3 | 9662 | Cell Signaling Technology | |
| Cleaved-caspase 3 | 9664 | Cell Signaling Technology | |
| PARP | 9532 | Cell Signaling Technology | |
| Cleaved-PARP | 5625 | Cell Signaling Technology | |
| Ki-67 | ab16667 | Abcam | |
