# Supplementary material for: Phosphoenolpyruvate carboxykinase 2-mediated metabolism promotes lung tumorigenesis by inhibiting mitochondrial-associated apoptotic cell death
Source: Front Pharmacol. 2024 Aug 9;15:1434988. doi: 10.3389/fphar.2024.1434988 (PMC11347759; doi:10.3389/fphar.2024.1434988)
Supplement: Supplementary file 3 [file Table3.DOCX]

| Supplementary Table 3. Sequences of siRNA and shRNA. | | |
| --- | --- | --- |
| Gene Name | Sense(5’-3’) | Antisense(5’-3’) |
| ATF4-Control | UUCUCCGAACGUGUCACGUTT | ACGUGACACGUUCGGAGAATT |
| ATF4-sequence1 | CUCCCAGAAAGUUUAACAATT | UUGUUAAACUUUCUGGGAGTT |
| ATF4-sequence2 | GUGAGAAACUGGAUAAGAATT | UUCUUAUCCAGUUUCUCACTT |
| PCK2-Control | UUCUCCGAACGUGUCACGUdTdT | ACGUGACACGUUCGGAGAAdTdT |
| PCK2-Sh1 | GGUGAUUGUAACUCCUUCUTT | AGAAGGAGUUACAAUCACCTT |
| PCK2-Sh2 | GGCAGAUAUUGAGAGAAUATT | UAUUCUCUCAAUAUCUGCCTT |
